# Empyema of the Accessory Sinuses

**Published:** 1915-06

**Authors:** J. Calhoun McDougall

**Affiliations:** Atlanta, Ga.; Member Alumni Association, New York Eye and Ear Infirmary. Assistant Oculist and Aurist to the Grady Hospital, Atlanta, Ga.


### EMPYEMA OF THE ACCESSORY SINUSES.

By J. Calhoun McDougall, M. D., Atlanta, Ga.

Member Alumni Association, New York Eye and Ear Infirmary. Assistant Oculist and Aurist to the Grady Hospital, Atlanta, Ga.

There has been an epidemic of LaGrippe in our city during the past winter there has been a large number of sinus infections, the influenza bacillus seeming to have a peculiar fondness for lurking in these cavities. In this group are included the ethmoidal, frontal, sphenoidal and maxillary sinuses.

The empyema may be classified as acute or chronic depending upon the duration of infection.

Acute empyema seldom involves one of this group alone except when an antrum is infected from a diseased tooth. The most common causes are acute rhinitis, influenza, pneumonia, measles, scarlet fever, diptheria, typhoid fever and other infective diseases. In the vast majority of these cases there is some abnormal condition in the nose such as deviations of the septum, polypi, cystic conditions of the turbinates, congentially narrow nasal cavities and marked hypertrophies of the nasal mucous membrane. The antrum often becomes the reservoir for pus from the frontal and ethmoids without its mucous membrane becoming markedly inflamed. Infected roots of the bicusped or molar teeth, syphilitic necroses and malignant disease occasionally induce an acute empyema in the antrum of highmore.

The symptoms of acute empyema of these sinuses are pain and discharge. The pain may be localized over the cavity mostly infected, it may be referred or general. It may be slight or severe dependent upon the amount of pus and the obstruction to drainage. The former varies in amount with the size of the cavity involved. In the earlier stages it is thick whitish muco pus, later it becomes thinner yellowish pus and often has a foul odor. The temperature may be slightly or greatly elevated depending upon the nature of infection and the disease which it follows. We usually get acute tenderness on pressure over the bony cavity and transillumination usually shows a darkened area; often a very thick bony wall of antrum or sinus gives a dark shadow which is deceptive to the rhinologist.

It is important to recognize these symptoms early so that less radical methods can be used to establish drainage and relieve the patient of long suffering and the establishment of a chronic empyema.

The treatment of these cases consists in the evacuation of pus from the sinuses. If there is any obstruction in the nasal passages this must be removed. In most cases of hyertrophy of the turbinates or nasal mucosa, a spray of adrenalin and cocain solution or warm alkaline douches properly administered produce drainage. A patient should always be kept in a position best for the drainage of a particualr sinus. In the frontal, ethmoidal and sphenoidal suppurations they should be in an upright sitting posture and with the antra involved they should lean the head well forward in a horizontal position, resting upon the opposite side. The foramen for the natural drainage of the antrum is located at the top of the lateral wall and serves better for a proof in Darwin's theory of evolution than it does for draining this cavity in man. I think we should be as conservative as possible in our surgical proceedures in acute empyema. It is often necessary to snare off the anterior third of the middle turbinate or remove polypi for drainage of the ethmoids or frontal and in time if this does not relieve symptoms the naso-frontal duct may be enlarged but there are rare ocacsions that demand a Killian Radical operation.

The lateral wall of the antrum should be punctured with a curved trocar and canula about one inch posteriorly under the inferior turbinate and irrigated with a mild antiseptic solution. This is done under local anesthesia with little pain and the antrum usually clears up after a few irrigations. If it does not subside after three or four irrigations it is best to make a larger opening in the same place with a chisel punch about three-eights of an inch in diameter; this will remain open and the patient can irrigate the cavity twice a day by inserting a silver or hard rubber catheter through this opening and the pus will also drain out if it forms rapidly. This method does not necessitate the loss of a tooth and does not give the patient as much pain as the old method still in use by dental surgeons. In cases where the antrum is directly infected from a diseased tooth it is better to make the opening for irrigation through the root or facet of that tooth.

The sphenidal sinus may be opened by making a puncture through the normal opening in the superior meatus, this requires very careful tecnique as the bony walls of this sinus are very thin and there is a possibility of opening into the cranial cavity with serious complications. As the optic nerve passes over the roof of this sphenoidal sinus softening of the bone from closed empyema or trauma from a curette often produces an optic neuritis which may result in atrophy and blindness.

Rest in bed with usual medical treatment is necessary in these infections. I have found the use of vaccines very efficient in acute empyema of the accessory sinuses, especially when the influenza bacillus is predominant.

It is the chronic empyema of the accessory sinuses that is most entertaining to the rhinologist. As the diagnosis is more difficult and the treatment more radical they come to him after years of neglect with complications of auto-intoxication, gastro-intestinal disorders, general breakdown of the nervous system accompanied by constant neuralgia and headaches. Chronic empyema is invariably secondary to an acute infection which has not been properly treated. The sinuses are usually involved separately but occasionally a chronic ethmoiditis will infect a frontal or antrum or visa versa. The early stages are the same as those in acute empyema; later the mucous membrane lining the cavities forms into a thick polypoid mass, often with large polypi projecting out. When the later are seen in the nasal cavity it usually indicative of chronic suppuration from one of the sinuses.

The symptoms characterizing chronic empyema are the constant discharge of pus with foul odor in the nose or pharynx, increasing during the presence of frequent colds and a fullness in the forehead, dull headache in the occiput, neuralgic pains in the face but no sharp constant pain. Patients complain of having so-called catarrh. Occasionally blindness results from an optic neuritis due to toxic absorption. Some complications of the general condition are usually present. Temperature is rare. The methods of examination and diagnosis are the same as in acute empyema.

The surgical treatment is far more radical. The nasal cavity should be made free by a submucous resection of the septum if there is a marked deflection or spurs. Snare out all polypi and remove diseased adenoids and tonsils. If suppuration continues after free drainage is established and surgical treatment used as I described in the acute conditions for a suitable length of time then a radical operation is indicated on the sinus in volved. After any of the radical operations a patient should be kept under observation for a long time to assure best results. I have not found vaccine treatment of any appreciable assistance in the treatment of chronic empyema in the accessory sinuses.

I have covered this broad subject very briefly, but I want to stress the importance to physicians in general practice of considering these discourses in determining their diagnosis of the various malidies that are predisposed by empyema of the accessory sinuses of the nose.

J. Calhoun McDougall, M. D.

1304 Empire Bldg.

